# Age biases the judgment rather than the perception of an ambiguous figure

**DOI:** 10.1038/s41598-021-88139-1

**Published:** 2021-04-21

**Authors:** Ambroos Brouwer, Xuxi Jin, Aisha Humaira Waldi, Steven Verheyen

**Affiliations:** Department of Psychology, Education and Child Studies, Erasmus University Rotterdam, Post Box 1738, 3000 DR Rotterdam, The Netherlands

**Keywords:** Psychology, Human behaviour

## Abstract

Older participants who are briefly presented with the ‘my wife/mother-in-law’ ambiguous figure estimate its age to be higher than young participants do. This finding is thought to be the result of a subconscious social group bias that influences participants’ perception of the figure. Because people are better able to recognize similarly aged individuals, young participants are expected to perceive the ambiguous figure as a young woman, while older participants are more likely to recognize an older lady. We replicate the difference in age estimates, but find no relationship between participants’ age and their perception of the ambiguous figure. This leads us to conclude that the positive relationship between participants’ age and their age estimates of the ambiguous ‘my wife/mother-in-law’ figure is better explained by the own-age anchor effect, which holds that people use their own age as a yard stick to judge the age of the figure, regardless of whether the young woman or the older lady is perceived. Our results disqualify the original finding as an example of cognitive penetrability: the participants’ age biases their judgment of the ambiguous figure, not its perception.

## Introduction

Ambiguous figures are figures that contain two or more different images and therefore can be perceived in distinct ways. The classic duck-rabbit ambiguous figure^1^, for instance, contains an image of a rabbit facing right and an image of a duck facing left. People see only one of these images at a time. Ambiguous figures create amusing visual effects and are therefore often found in popular media to attract and entertain readers. However, they are also used in psychological experiments to study the role of both conscious and subconscious processes in their perception^2–5^.

**Figure 1:**
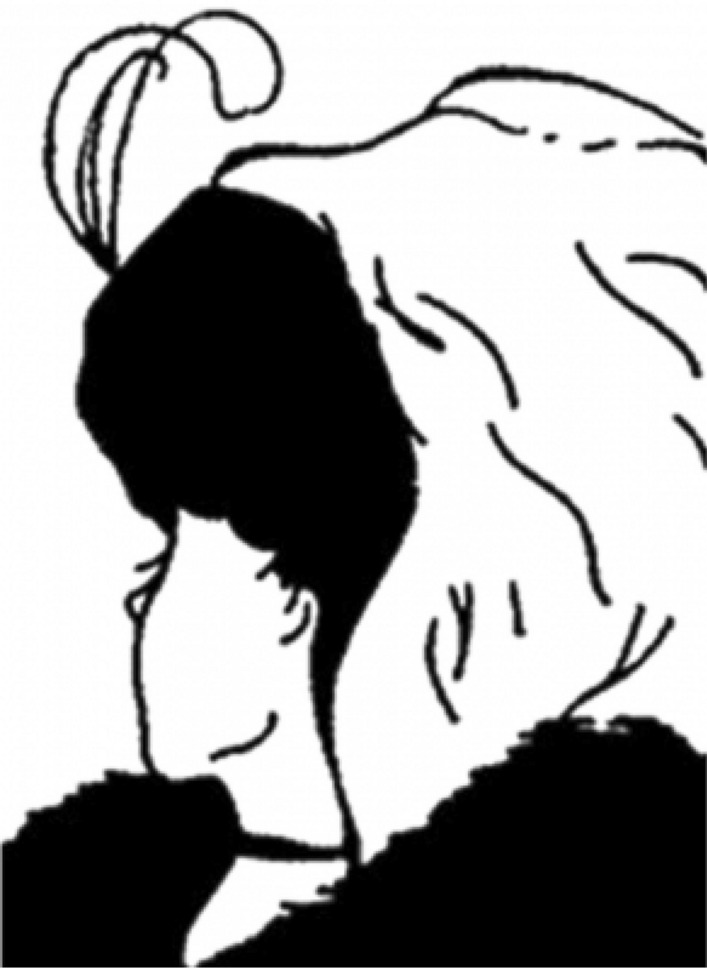
My Wife and My Mother-In-Law, by the cartoonist W. E. Hill, 1915. This media file is in the public domain in the United States. This applies to U.S. works where the copyright has expired, often because its first publication occurred prior to January 1, 1923.

Nicholls, Churches, and Loetscher^6^ used an ambiguous figure to investigate whether high-level social processes subconsciously affect face perception. Following Bar^7^, they hypothesized that early visual brain regions pass on a partially analyzed version of a face to the prefrontal cortex where it is subject to social expectations that may influence the percept that is formed in the temporal cortex and any subsequent behavior that is directed at it. To test this hypothesis, Nicholls et al. presented their participants with the ambiguous ‘my wife/mother-in-law’ figure (Fig. 1). This figure, which was originally introduced as a psychological tool by Boring^8^, can be perceived as either a young woman facing away from the observer, or as an older lady facing more toward the observer. After a presentation time of 500 ms, which was deemed too short for conscious processes to be of influence, participants were asked to estimate the age of the woman they saw. Nicholls et al. found that older participants estimated the woman in the figure to be significantly older than young people did. On average, participants who were older than 30 estimated the woman to be 6.32 years older than did participants who were 30 years or younger. Nicholls et al. also established a reliable positive relationship between the estimated age and the participants’ own age. Across 393 participants, the correlation between the two variables measured 0.24.

The findings by Nicholls et al. suggest that the way a face is processed is subconsciously affected by the perceiver’s age. But how does one’s age influence face perception exactly? Nicholls and colleagues interpret their finding in terms of a social group bias. The in-group vs. out-group distinction is a common one in the social sciences. One’s in-group consists of people with who one shares similar characteristics. These characteristics are not shared with members of the out-group. Studies have found that people exhibit a recognition bias toward in-group members. That is, people are better able to recognize individuals of the same ethnicity^9^, gender^10^, or age^11,12^. For example, Wright and Stroud^13^ showed that an eyewitness is more accurate at identifying the suspect of a crime if they belong to the same age group. The increased recognition accuracy for similarly aged faces, also known as the own-age bias in face perception, is believed to be due to increased contact and familiarity with people of one’s own age group^14–16^, as well as more in depth processing of the faces of the members of one’s age in-group^17–20^. According to a meta-analysis on the own-age bias in face recognition^21^, the way participants study and encode faces does not influence the size of the group difference, suggesting that the bias presents automatically, which is in line with the unconscious age effects on face perception observed by Nicholls and colleagues.

If superior recognition of faces from one’s own age group is at the basis of the finding by Nicholls et al., this would imply that an unconscious social group bias led participants to recognize the woman in the ambiguous ‘my wife/mother-in-law’ figure who most closely matches their age group, resulting in a higher age estimate by older than by young participants. That is, participants would perceive the ambiguous figure differently, depending on their age. Young participants would be likely to see the young woman facing away from the observer, while older participants would be more inclined to perceive the older lady facing the observer. This would constitute a clear example of cognitive penetrability, where higher-level cognitive processes influence what we see^22,23^. Such top-down effects on visual perception have far-reaching consequences in that they challenge the traditional modular view on perception and cognition^24,25^.

A second interpretation does not require the figure that is seen by the participants to differ systematically between age groups. What Nicholls et al. observed might be explained alternatively by the own-age anchor effect. According to Tversky and Kahneman^26^, anchor effects occur when people’s estimates assimilate toward a salient value (the anchor) causing their estimates to be inaccurate. In the case of the own-age anchor effect, people use their own age as an anchor, causing age estimates to be biased toward their own age. An early study on the own-age anchor effect conducted by Mintz^27^, for example, demonstrated that children estimate the age of the cartoon character Peter Pan to be similar to their own age. The observation that older participants estimate the ambiguous ‘my wife/mother-in-law’ figure to be older than younger participants do, could thus be the result of assimilation of the age estimates to participants’ own age, regardless of the woman that is perceived. That is, both when viewing the young woman and the old lady, participants would make an age estimate that is anchored to their own age, resulting in a mean estimate difference between age groups, as well as a positive correlation between participant age and estimated age.

People’s tendency to assimilate others’ characteristics to their own has received less attention than the own-age social group bias, but empirical evidence supporting the own-age anchor effect is increasing^28–32^. The basis for the effect is not yet well understood. One reason why one’s own age is selected as a yardstick might be because people usually choose what is highly accessible in memory as their standard^33^. In that case, the own-age anchor effect might have a similar origin as the own-age bias in face recognition: both biases could result from increased familiarity with people of one’s own age^34^. Alternatively, the effect may be the result of a social cost reduction strategy, whereby participants overestimate the age of younger faces and underestimate the age of older faces in order to err on the safe side^32^. The own-age anchor effect is also reminiscent of egocentricity biases, whereby people consider themselves rather than others as a reference point^35^, making them judge others to be more similar to themselves than the other way around^36^ or project their own properties onto others^37^. Anchoring estimates to salient or normative values might be particularly adaptive in highly uncertain situations, where there is insufficient other information to base one’s judgment on^34^. The brief presentation of an ambiguous figure might therefore constitute a situation in which the own-age anchor effect is likely to present.

The own-age social group bias is different from the own-age anchor effect in that the former is usually considered to be a perceptual bias, while the latter tends to be characterized as a cognitive bias. Extensive experience with faces of our own age group is believed to facilitate their perceptual processing according to the own-age social group bias, while in the case of the own-age anchor effect it is believed to skew our judgments. The own-age social group bias thus predicts that young and older participants are respectively more inclined to see the young woman and the older lady in the ambiguous ’my wife/mother-in-law’ figure, and that this difference in perception is what is driving the higher age estimates by older than by young participants. Were the relationship between own age and age estimates due to the own-age anchor effect, the ambiguous figure would not need to be perceived systematically different by young and older participants. Participants would assimilate the age of the perceived women to their own, regardless of whether the young woman or older lady is perceived. The latter would disqualify the finding by Nicholls et al. as an example of cognitive penetrability in that the age of the participants would affect their judgments of (the age of) the ambiguous figure, not their perception of the figure. While top-down effects on visual perception are incompatible with the modular view on perception and cognition, top-down effects on judgment are commonplace and therefore have less far-reaching consequences for the way we conceive of the mind^38^.

We present a study that aims to examine whether the findings by Nicholls et al. can be best explained by the own-age social group bias or the own-age anchor effect. In order to do this, we will replicate the original study as closely as possible, but in addition ask participants to indicate whether they saw the young woman or the older lady. This way, participants’ percepts do not have to be inferred from their age estimates. It allows us to establish whether participants’ age biases their perception or their judgment of the ambiguous figure^38^. If participants’ age were to affect their perception of the ‘my wife/mother-in-law’ figure, this would constitute evidence that older participants’ higher age estimates of the ambiguous figure result from an own-age social group bias and the age difference would constitute evidence for a top-down effect on perception. If not, the own-age anchor effect better explains the difference in the estimates of the ambiguous figure’s age, which would then not be indicative of cognitive penetrability of perception.

## Methods

This experiment was conducted in accordance with the Code of Ethics for Research in The Social and Behavioural Sciences Involving Human Participants of the Erasmus University Rotterdam. The study was considered exempt by the Ethics review Committee of the Department of Psychology, Education and Child Studies at Erasmus University Rotterdam. It was pre-registered on the Open Science Framework (see osf.io/xqc35/). The materials are also available there (osf.io/y3bqa/).

### Participants

An a priori power analysis was conducted using the R package pwr^39^ to determine the required sample size to test the mean difference in age estimation between two independent groups using a one-tailed test with an *α* of 0.05, and assuming the original effect size (Cohen’s *d* = 0.39). Results showed that a sample of 232 participants, comprised of two equally sized groups of *n* = 116, was required to achieve a power of 0.90. In order to compensate for the potential exclusion of participants, a total of 260 participants was recruited. An analysis of participants’ eligibility for inclusion (see below for criteria) resulted in the exclusion of 14 participants, reducing the final sample to *N* = 246.

All participants were adult US citizens (*f* = 135, *m* = 111) recruited using Prolific Academic and compensated with $0.30. In order to make sure that the young and older group had a similar number of participants, the survey was run twice; once for people of 30 years or younger, and once for people older than 30. The mean age of the young group was 23.73 years (*SD* = 3.28, *n* = 124), while the mean age of the older group was 44.98 years (*SD* = 12.35, *n* = 122). All participants answered demographic questions regarding their age, sex and nationality. The experiment took on average 83.55 s to complete.

### Materials and procedure

The experiment was set up to approximate the original study by Nicholls et al. as closely as possible. There are three noteworthy deviations from the original. After estimating the age of the woman in the ‘my wife/mother-in-law’ figure, participants were presented with two additional figures highlighting the young woman and older lady to have them indicate which of the two images they perceived. Participants were also asked whether they had seen the ambiguous figure prior to the experiment and invited to estimate the age of a computer generated face. The latter two changes were respectively included to explore the effect of familiarity with the ambiguous figure on age estimation and to establish the generalizability of the relationship between participant age and age estimates.

The experiment was implemented in the software Qualtrics version May 2020 (Qualtrics, Provo, UT). After providing informed consent and indicating their age, sex, and nationality, participants were shown a screen which asked them to pay close attention to the figure on the next screen, as it would only be shown for a short period of time. After clicking to the next page, a copy of the original ‘my wife/mother-in-law’ figure was shown for 500 ms (Fig. 1). Subsequently, participants’ eligibility was tested using two questions. First, participants were asked to indicate if they saw a person, an animal, or neither. Participants who responded ‘person’, were then asked if that person was male or female. When a participant answered one of these questions incorrectly, the experiment was terminated immediately. Otherwise, participants indicated whether they had seen the figure prior to the experiment, after which they were asked to estimate the person’s age in whole numbers.

**Figure 2:**
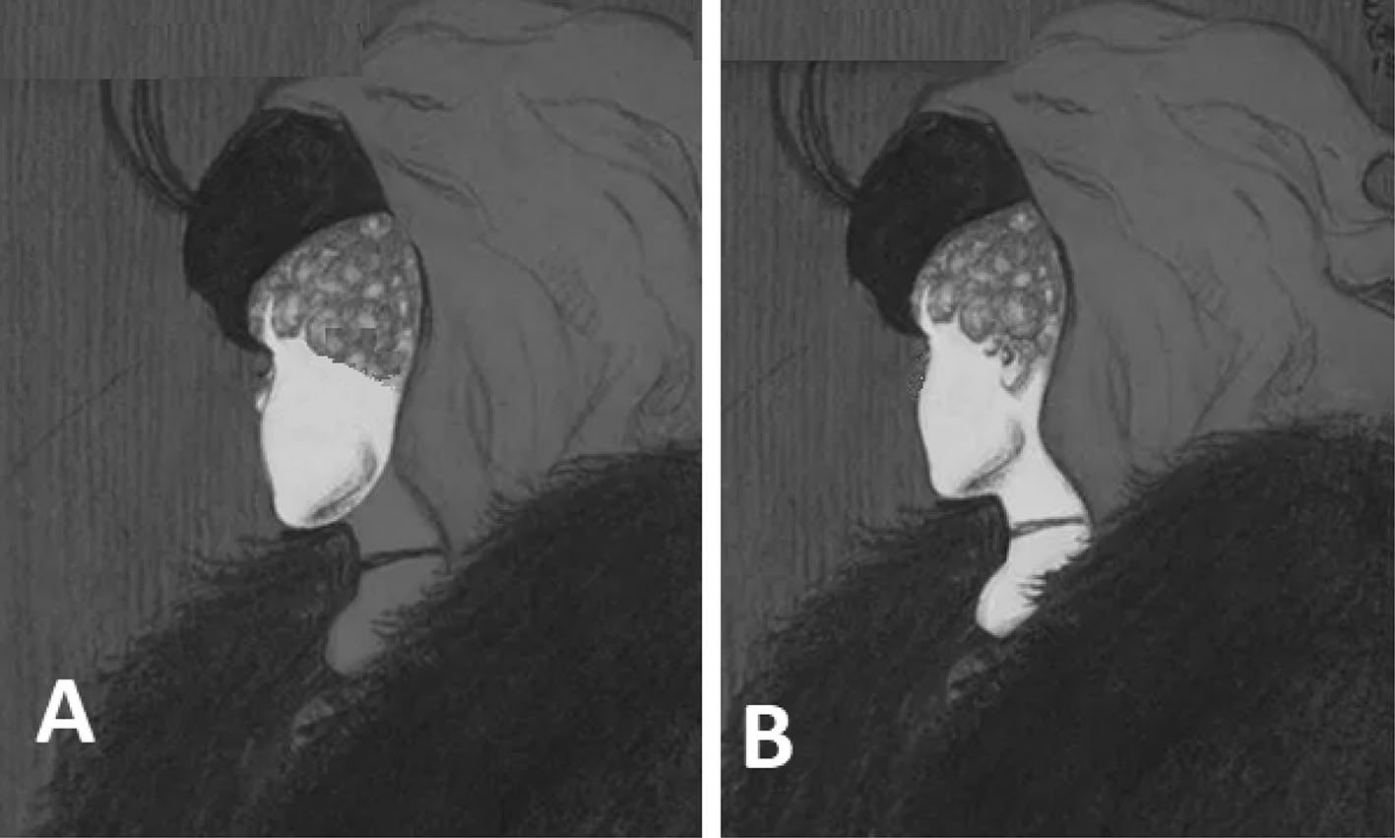
Figure highlighting the young woman (**A**) and the older lady (**B**) after Shakhnazarova (2018).

A text was then shown that stated that the figure consisted of two different women. The participants were told that on the next screen, two figures would be shown, each highlighting one of these two women (Fig. 2). The order in which the two percepts were highlighted in the figure was counterbalanced across all participants. That is, for half of the participants, the young woman was depicted in the left panel (panel A), while for the other half, the young woman was depicted in the right panel (panel B). Participants were asked to indicate which of the two women they had seen. Based on a study by Georgiades and Harris^40^, critical features were removed or highlighted in order to enable participants to discriminate clearly between the two percepts. From panels A and B in Fig. 2, for instance, the eye of the older lady, and the nose of the young woman were removed, respectively. These modified figures were originally presented by Shakhnazarova in the newspaper The Sun^41^.

After completing this part of the experiment, participants were told once again to pay attention to the figure on the next page, as it would only be presented for a short period. This time, a computer-generated face was shown for 500 ms. The face was synthesized using the FaceGen Modeller Software (Singular Inversions, 1998) to represent an average thirty year-old Caucasian female. The participants were once more asked to estimate the age of the face in whole numbers, after which they were thanked for participating.

## Results

All analyses were performed with the statistical software R version 3.6.1^42^ using the packages BayesFactor^43^, dplyr^44^, ggthemes^45^, ggplot2^46^, gridExtra^47^, lsr^48^, and sjstats^49^. An *α* = 0.05 was employed in all analyses. The data and the R script are available on osf.io/y3bqa/. We start by reporting the results of the pre-registered confirmatory analyses pertaining to the own-age social group bias and the own-age anchor effect. We then turn to the results of the pre-registered exploratory analyses looking into the effect of familiarity with the ambiguous figure. The results of the analyses pertaining to the computer-generated face are supplied as Supplementary Information, along with additional visualizations and analyses requested by the reviewers.

### Primary analyses: replication of original findings

In order to replicate the original findings of Nicholls et al.^6^, a one-tailed independent samples t-test was performed to examine the difference in mean age estimates of the ambiguous figure between the age groups. Older participants were found to estimate the ambiguous figure significantly older (*M* = 41.04, *SD* = 17.51) than young participants (*M* = 33.50, *SD* = 14.56); *t*(234.76) = 3.67; *p* < 0.001, Cohen’s *d* = 0.47. On average, older participants thus estimated the person in the ‘my wife/mother-in-law’ figure to be 7.54 years older than young participants did. A one-tailed Pearson correlation was computed between participants’ own-age and their age estimates of the ambiguous figure. Results indicated that there was a significant positive association between participants’ age and their age estimates; *r*(244) = 0.22, *p* < 0.001. Similar results were obtained in the original experiment of Nicholls et al., who found a mean age difference of 6.32 years and a correlation of 0.24.

In the original paper, the initial analysis was repeated with the 10% youngest and 10% oldest participants. In the case of the current experiment, this would cause some 21-year old and some 57-year old participants to be excluded from the analysis on an arbitrary basis. Therefore, 8.94% of the youngest and oldest participants were selected for the analysis. This resulted in a sample of *n* = 44 with 22 participants in each age group. The youngest participants had a mean age of 19.14 years (*SD* = 0.83) and the oldest participants had a mean age of 65.77 (*SD* = 5.45). A one-tailed independent samples t-test showed the mean age estimate of the ambiguous figure to differ significantly between the youngest (*M* = 29.64, *SD* = 10.78) and the oldest participants (*M* = 43.41, *SD* = 17.26); *t*(35.227) = 3.17, *p* = 0.002, Cohen’s *d* = 0.96, demonstrating that the results are not a consequence of the arbitrary age split, and that the difference in the mean age estimates was 6.24 years greater when comparing the oldest with the youngest participants instead of the original age groups.

### Primary analyses: social group bias or anchor effect?

Participants were assigned to two different percept groups based on whether they reported seeing the young woman or the older lady. The older lady was perceived by the majority of the participants. Only 114 out of 246 participants (46.34%) reported perceiving the young woman. Another one-tailed independent samples t-test was performed to compare the mean difference in the estimated age of the ambiguous figure by the young percept group (*M* = 32.89, *SD* = 13.24) and by the old percept group (*M* = 41.00,* SD* = 18.09). This difference was found to be significant: *t*(237.81) = 4.05,* p* < 0.001, Cohen’s *d* = 0.51, indicating that the older lady was perceived to be significantly older than the young woman. This difference is to be expected if participants can reliably indicate which of the two clearly differently aged women they perceived in the ambiguous picture.

A chi-square test was performed to determine whether the frequency of the reported percepts differed between the age groups. The relationship was not significant (*χ*^2^(1) = 0.01, *p* = 0.91). Because lack of a difference in perception between age groups is crucial to argue that there is no top-down effect on visual perception, we followed up the chi-square test with its Bayesian equivalent to establish the evidence in favor of the null hypothesis of no dependence between age group and percept group. (The reported Bayesian analyses were not pre-registered, but suggested by a reviewer.) Using an independent multinomial sampling plan, we obtained a Bayes factor of 6.29 to 1 in favor of the null suggesting that participants’ age does not systematically influence how they perceived the figure. Therefore, the relationship between participants’ own age and their age estimates of the ambiguous figure does not appear to be a consequence of the own-age social group bias.

To determine whether the relationship between own age and estimated age was independent of the woman that was perceived in the ambiguous figure, a one-tailed independent samples t-test was conducted for each percept group independently. A significant difference in age estimates was found within the young percept group between the young (*n* = 57, *M* = 28.91, *SD* = 8.90) and the older participants (*n* = 57*, M* = 36.86, *SD* = 15.56); *t*(89.135) = 3.35, *p* < 0.001, Cohen’s *d* = 0.63. This difference between the young participants (*n* = 67, *M* = 37.40, *SD* = 17.16) and the older participants (*n* = 65*, M* = 44.71, *SD* = 18.41) was also found within the old percept group; *t*(128.7) = 2.36, *p* = 0.01, Cohen’s *d* = 0.41.

**Figure 3:**
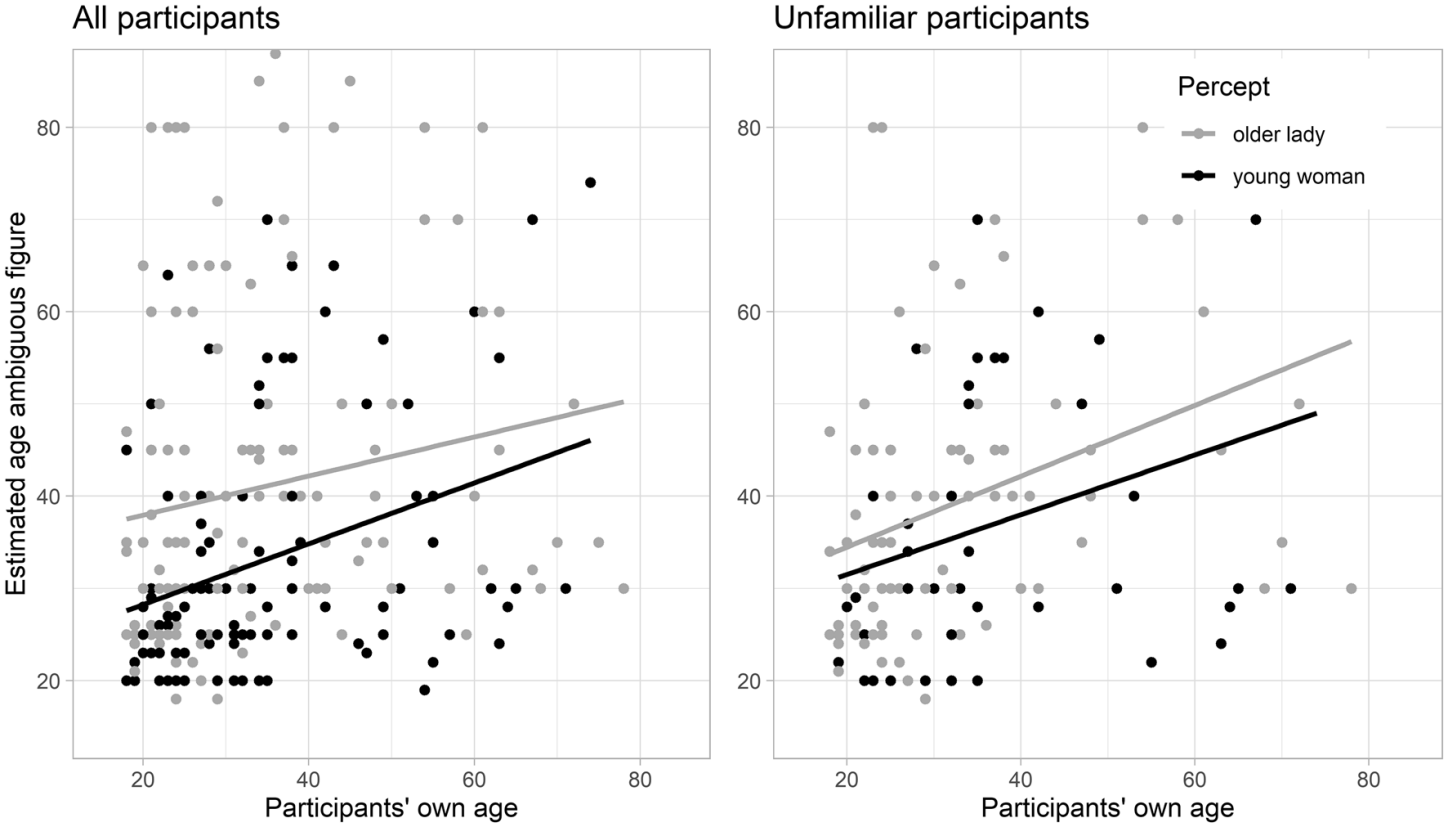
Relationship between participants’ own age and the estimated age of the ambiguous ‘my wife/mother-in-law’ figure as a function of the two percepts (young woman = black; older lady = gray). The left panel depicts the data from all participants (*N* = 246). The right panel only depicts the data from participants who reported not having seen the ambiguous ‘my wife/mother-in-law’ figure prior to the study (*n* = 126).

A one-tailed Pearson correlation between participants’ own age and the age they estimated the ambiguous figure to be was calculated for each of the percept groups. The left panel of Fig. 3 summarizes the results. It shows a significant positive association between participants’ own-age and their age estimates within both percept groups; *r*(112) = 0.34, *p* < 0.001 in the young percept group (black), and *r*(130) = 0.17, *p* = 0.03 in the old percept group (gray). That is, participants provided higher age estimates for the ambiguous figure the older they were, regardless of whether they perceived the young woman or the older lady.

Finally, a two-way ANOVA was carried out to investigate simultaneously the effect of age group and percept group on the estimated age of the ambiguous figure. The interaction of age group and percept group was not significant (*F*(1,242) = 0.03, *p* = 0.87, *ω*^2^ = 0.00). Age group (*F*(1,242) = 14.32, *p* < 0.001, *ω*^2^ = 0.05) and percept group (*F*(1,242) = 16.73, *p* < 0.001, *ω*^2^ = 0.06) had independent significant effects on age estimation.

Taken together, these results provide support for the hypothesis that the positive relationship between participants’ own age and their age estimates of the ‘my wife/mother-in-law’ ambiguous figure are best explained by the own-age anchor effect.

### Secondary analyses: familiarity with the ambiguous figure

After watching the ‘my wife/mother-in-law’ figure for 500 ms, participants indicated whether they had been exposed to the ambiguous figure prior to the experiment. In order to determine the effect of familiarity on the perception of the ambiguous figure, a chi-square test was conducted. A significant relationship between familiarity (prior exposure/no prior exposure) and the image the participants reported seeing (young woman/older lady) was found: *χ*^2^(1) = 13.55, *p* < 0.001. Seventy of the 120 participants who were familiar with the ambiguous figure perceived the young woman (58.33%), whereas only 44 of the 126 participants who were unfamiliar with the figure perceived the young woman (34.92%). It thus appears that the young lady is more likely to be perceived in the ‘my wife/mother-in-law’ figure by participants who have been exposed to the ambiguous figure before.

When researchers aim to study the processes involved in the perception of ambiguous figures, they are presumably interested first and foremost in the manner in which participants who are confronted with these figures for the first time process them. We therefore repeated the above analyses for the participants who had no prior experience with the ‘my wife/mother-in-law’ figure to assess whether the reported findings are representative for this subgroup. When interpreting these results, one should take into account that the power of these analyses is expected to be lower than those in the main article since they are conducted on a subsample of the participants (*n* = 126 compared to *N* = 246). Note that participants’ familiarity with the ambiguous figure was independent of their age-group membership (*χ*^2^(1) = 0.02, *p* = 0.90). The corresponding Bayes factor, obtained using an independent multinomial sampling plan, is 6.26 to 1 in favor of the null. Hence, the unfamiliar participants were equally distributed across both age groups.

A one-tailed independent samples t-test again indicated a significant difference in the age estimates by the young (*n* = 63, *M* = 33.65, *SD* = 13.35) and the older participants (*n* = 63, *M* = 43.62, *SD* = 15.83); *t*(120.55) = 3.82, *p* < 0.001, Cohen’s *d* = 0.68. Among the participants with no a priori exposure to the ‘my wife/mother-in-law’ figure, the one-tailed Pearson correlation between participant age and estimated age was found to be significant (*r*(124) = 0.32, *p* < 0.001). The main findings by Nicholls et al. thus also hold when the analyses are restricted to the participants who had no prior experience with the ambiguous figure.

Another chi-square test was conducted to investigate the relationship between age group membership (young/older) and the reported percepts (young woman/older lady) among participants who were unfamiliar with the ambiguous figure (*χ*^2^(1) = 3.49, *p* = 0.06). The corresponding Bayes factor obtained using Poisson sampling is 2.31 to 1 in favor of the alternative hypothesis of age group-percept group dependence. We defer a debate as to whether or not this constitutes statistical evidence for a relationship between age and percept group to the discussion, and instead focus here on the observation that 46 of 63 people in the young group (73.02%) reported seeing the older lady, whereas 36 of 63 people in the older group (57.14%) reported seeing her. This data pattern does not agree with an interpretation in terms of the own-age social group bias, as this would predict that the older lady is more likely to be perceived by older participants than by young participants; not the other way around.

To determine whether the relationship between own age and estimated age was also independent of the woman that was perceived among participants who were unfamiliar with the ‘my wife/mother-in-law’ ambiguous figure, a one-tailed independent samples t-test was conducted for each percept group independently. A significant difference in age estimates was found within the young percept group between the young (*n* = 17, *M* = 30.06, *SD* = 9.47) and the older participants (*n* = 27*, M* = 41.56, *SD* = 16.52); *t*(41.744) = 2.93, *p* = 0.003, Cohen’s *d* = 0.81. This difference between the young participants (*n* = 46, *M* = 34.98, *SD* = 14.38) and the older participants (*n* = 36*, M* = 45.17, *SD* = 15.35) was also found within the old percept group; *t*(72.874) = 3.07, *p* = 0.002, Cohen’s *d* = 0.69.

To assess whether the own-age anchor effect can be used to interpret these differences, we calculated the Pearson correlation between unfamiliar participants’ own age and their age estimates, for each of the percepts. The right panel of Fig. 3 shows that the positive association between participants’ own-age and their age estimates holds for both the young percept group (*r*(42) = 0.32, *p* = 0.02, black) and the old percept group (*r*(80) = 0.35, *p* = 0.001, gray).

In sum, among participants with no prior exposure to the ‘my wife/mother-in-law’ figure, the own-age anchor effect appears to be present both in participants reporting seeing the young woman and participants reporting seeing the older lady. In other words, the findings from the main analysis are not driven by participants who are familiar with the ambiguous figure due to prior exposure, and can therefore not be attributed to demand characteristics.

## Discussion

One’s age has been shown to affect how old one estimates the ‘my wife/mother-in-law’ ambiguous figure to be^6^. In this study, we replicated the finding that older participants estimate the woman in the figure to be significantly older than young participants do. Contrary to what was originally suggested, we take this age difference to reflect the own-age anchor effect, not a social group bias toward processing similarly aged faces.

If the difference in age estimation were due to the social group bias, we would expect a propensity among older participants to report seeing the older lady and a propensity among young participants to report seeing the young woman. We did not find this to be the case. The proportion of times the wife and the mother-in-law were reported was comparable in the two age groups. This does not exclude the possibility that there were systematic age differences in the manner in which the ambiguous figure was processed, however (see, for instance^50^). It might just be that these processing differences do not result in a difference in the reported percept.

Our findings are more in line with the own-age anchor effect, according to which people tend to assimilate toward their own age, when estimating someone else’s age^28–32^. The own-age anchor effect does not require participants’ perception of the ambiguous figure to be influenced by their own age. It predicts a positive association between participants’ own age and their age estimates, irrespective of the woman they see. In other words, there should be a difference in age estimation between younger and older participants even if they perceive the same woman. This is what we found.

A final result that appears to be more in line with the own-age anchor effect than with the social group bias, is the observation that the age estimates for the ambiguous figure are rather low, even in older participants reporting seeing the older lady. The older lady in the ambiguous figure is supposedly intended to be older than the average age of 44.71 years reported by this group of participants with an average age of 45.66 years (*SD* = 12.51). It strengthens our opinion that participants’ age estimates are driven by a cognitive bias to use one’s own age as a yardstick, rather than high-level social processes subconsciously affecting face perception. The brief presentation of an ambiguous figure might just constitute the uncertain circumstances in which the own-age anchor effect is likely to occur. When people have an age judgment to make, but little information is available to base that judgment on, it is not illogical for them to anchor it on a normative value such as their own age, which is salient and likely to be representative for the majority of people they interact with.

Our results disqualify the original finding by Nicholls et al.^6^ as an example of cognitive penetrability. The absence of an age difference in the reported percept for the ‘my wife/mother-in-law’ figure indicates that the age difference in age estimation of this ambiguous figure does not reflect a top-down effect of cognition on visual perception. Rather than challenging the modular view on the organization of our cognitive and perceptual faculties, it is better explained as a judgment bias. It is easy to see how the latter bias could have been overlooked. Based on relatively higher age estimates, it is tempting to infer that the ages of the percepts that form the basis of the judgments must differ as well. However, as Firestone and Scholl^38^ have convincingly shown for a number of studies that allegedly demonstrated top-down effects on perception, it is important to establish what participants actually saw, not infer it from their verbal reports. When we did so in our replication of Nicholls et al., it led us to conclude that participants’ age biases their judgment of the ‘my wife/mother-in-law’ ambiguous figure, not its perception.

While we interpreted the observation that older participants estimate the ‘my wife/mother-in-law’ ambiguous figure to be older than young participants do in terms of the own-age anchor effect, we do not want to contend that it is the only factor determining age estimates. We believe that social influences might play a role as well. Specifically, people might only assimilate others’ characteristics to their own, if these others are considered part of the in-group. Sörqvist et al.^30^ showed that women demonstrate assimilation toward their own age when estimating the ages of male targets, but not vice versa. The authors speculated that this difference might result from men’s tendency to categorize others as an in-group or out-group member based on gender, while women do not. The findings by Sörqvist et al. would then be the result of women having larger age in-groups than men. That is, women’s age in-groups may include men, whereas men’s age in-groups do not include women. In the context of ambiguous figures, this could be further investigated by comparing the extent to which the own-age anchor effect presents in men and women’s age judgments of the ‘my wife/mother-in-law’ ambiguous figure, and a comparable ambiguous figure featuring differently aged men, such as the ‘my husband/father-in-law’ ambiguous figure^51^. In future work, both the mechanisms underlying the own-age anchor effect, as well as its ramifications should be further investigated. In the Supplementary Information, we report how we were unable to establish a reliable relationship between participants’ own age and their age estimate of a computer-generated face. This result shows that own-age anchor effects are not ubiquitous, which is important to take into account in light of their potential practical implications, for instance in the context of eyewitness testimonies and the provision of age-restricted services.

We found that the ‘my wife/mother-in-law’ ambiguous figure is well known by the general public. Almost half of the participants were familiar with it, and more importantly, participants’ familiarity with the ‘my wife/mother-in-law’ figure influenced how they perceived it, with the young woman being reported more frequently among participants who were already familiar with the ambiguous figure. Since one’s familiarity with the ambiguous figure affects how it is perceived, it might be warranted to look for ambiguous stimuli that have received less exposure in the popular media or to create new ones altogether. The current form of the ‘my wife/mother-in-law’ figure dates back to the 1930s, rendering the clothes of the women in the picture old-fashioned. One may question whether this makes for an ecologically valid stimulus to study the role of (un)conscious processes in age/face perception.

That being said, when we restricted the analyses to the participants who had no prior exposure to the ambiguous figure, older participants were still found to estimate its age to be higher than young participants did. Contrary to the predictions based on the social group bias, older participants did not report seeing the older lady more often than younger participants did. Irrespective of whether these participants perceived the young woman or the older lady, there was still a relationship between participants’ own age and their estimates of the age of the ambiguous figure. Taken together, these results for participants who had no a priori exposure to the ambiguous figure once more support an interpretation of the age difference in age estimation of the ambiguous figure in terms of the own-age anchor effect rather than in terms of a recognition bias toward age in-group members.

The data of participants who were unfamiliar with the ambiguous figure did provide the only piece of support for a tentative relationship between one’s age and one’s perception of the ‘my wife/mother-in-law’ ambiguous figure. The young woman was reported being seen by 27% of young participants compared to 43% of the older participants. A Bayesian chi-square test revealed a Bayes factor of 2.31 to 1 in favor of the alternative hypothesis of age group-percept group dependence compared to the null hypothesis of independence. After conducting the study, we learned about a similar result that was already obtained by Botwinick, Robin, and Brinsley in 1959^52^. They presented male participants with the ‘my wife/mother-in-law’ ambiguous figure and asked them to report what they saw within 90 s. Botwinick et al. found that of all participants who reported either of the percepts, 76% of the young participants and 94% of the older participants reported seeing the young woman. Both results only provide anecdotal evidence^53,54^ for the young woman percept being more prevalent among the older participants than among the young participants, but it is an intriguing finding nevertheless. At this point, we can only speculate as to the origin of this tentative difference. Previous research has shown that the interpretation of an ambiguous figure is dependent on the fixation point and the critical features of the image that are attended to^40,55^. Fixating a particular point of an ambiguous figure might make one process features that are critical for eliciting one interpretation of the figure, while fixating on other critical features will result in the other interpretation. Systematic age differences in gaze, for instance, might thus lie at its basis^50^. In order for the difference to qualify as a top-down effect of cognition on perception, proponents of cognitive penetrability would first have to come up with a compelling reason as to why older participants would, for instance, fixate a different set of critical features than young participants. If they managed to do that, the processing of the ‘my wife/mother-in-law’ ambiguous figure by novice young and older perceivers would make for a very interesting future study.

## Supplementary Information

Supplementary Information.

## Data Availability

The data that support the findings of this study are openly available on the Open Science Framework at osf.io/y3bqa/. The study was pre-registered (osf.io/xqc35). The data used in this article are licensed under a Creative Commons Attribution 4.0 International License (CC-BY), which permits use, sharing, adaptation, distribution, and reproduction in any medium or format, as long as you give appropriate credit to the original authors and the source, provide a link to the Creative Commons license, and indicate if changes were made. To view a copy of this license, visit http://creativecommons.org/licenses/by/4.0/.
