# Supplementary material for: Age biases the judgment rather than the perception of an ambiguous figure
Source: Sci Rep. 2021 Apr 21;11:8627. doi: 10.1038/s41598-021-88139-1 (PMC8060281; doi:10.1038/s41598-021-88139-1)
Supplement: Supplementary file 1 — Supplementary Information. [file 41598_2021_88139_MOESM1_ESM.docx]

Supplementary Information for

**Age Biases the Judgment Rather than the Perception of an Ambiguous Figure**

Ambroos Brouwer, Xuxi Jin, Aisha Humaira Waldi, & Steven Verheyen^*^

Erasmus University Rotterdam, Rotterdam, The Netherlands

*Correspondence should be addressed to Steven Verheyen,

Department of Psychology, Education and Child Studies, Erasmus University Rotterdam,

Post Box 1738 3000 DR Rotterdam, The Netherlands. E-mail: [verheyen@essb.eur.nl](mailto:verheyen@essb.eur.nl). https://orcid.org/0000-0002-6778-6744

This document contains additional visualizations and analyses that were requested during peer review, as well as the results of the pre-registered exploratory analyses (see osf.io/xqc35) pertaining to the generalizability of the own-age anchor effect to a computer-generated face. All analyses were performed with the statistical software R version 3.6.1 (R Core Team, 2016) and employed an *α* = .05. The data and the R script are available on osf.io/y3bqa.

1. Additional visualizations and analyses

Figure S1 depicts the distribution of the age estimates for the ‘my wife/mother-in-law’ ambiguous figure per percept group. It provides a summary representation of the data depicted in the left panel of Figure 3 in the main text. It too supports the finding reported in the main text that participants who reported seeing the old lady on average provide higher age estimates than the participants who reported seeing the young woman.

*
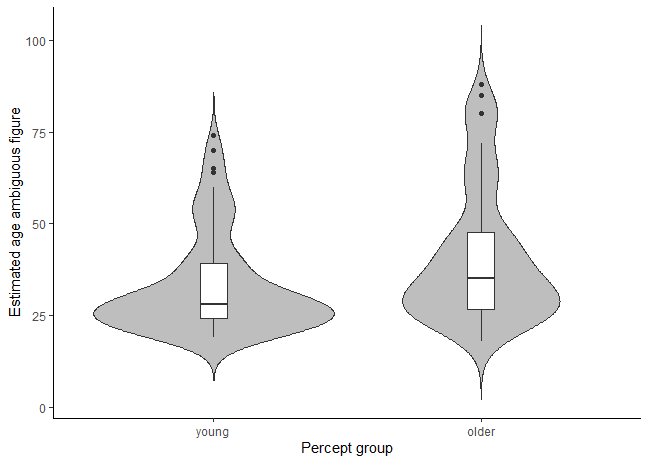
*

**Figure S1.** Violin plots depicting the distribution of age estimates for the ambiguous figure per percept group.

In the main text, we performed a two-way ANOVA to investigate simultaneously the effect of age group and percept group on the estimated age of the ambiguous figure. We found significant main effects of age group and percept group. The interaction of age group and percept group was not significant. The decision to operationalize participant age as the dichotomous variable age group followed the analyses by Nicholls et al. (2018) who were interested in establishing a social group bias.

If one were to discard this interest in a group bias and investigate the own-age anchor effect on its own, it is no longer necessary to operationalize participant age as a binary variable. A reviewer therefore suggested regressing the estimated age of the ambiguous figure on the binary variable percept group, the continuous variable participant age, and their interaction. A significant regression equation was found (F(3,242)=10.19, *p* < .001), with an *R^2^* of .11. In line with the results of the two-way ANOVA, we found significant effects of percept group and participant age, but not of their interaction. Participants who perceived the young woman estimated the ambiguous figure 12.08 years younger than participants who perceived the old lady, and a one-year increase in participant age corresponded to an increase of .21 years in the ambiguous figure’s estimated age.

1. Generalizability of the own-age anchor effect

In order to investigate the generalizability of the relationship between participant age and age estimates, we presented participants with an additional figure to estimate the age of. This figure was synthesized using the FaceGen Modeller Software (Singular Inversions, 1998) to represent an average thirty-year-old Caucasian female. The resulting face is presented in Figure S2. It too was presented for 500 ms, after which participants estimated its age in whole numbers.


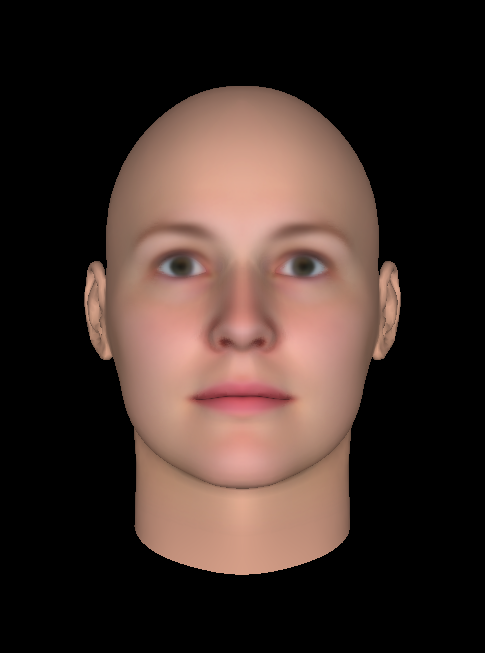


**Figure S2.** Computer-generated face representing an average thirty year-old Caucasian female. This figure was synthesized using FaceGen Modeller Core 3.18 (Singular Inversions, 1998, https://facegen.com/).

Figure S3 shows the relationship between the participants’ age and their age estimates for the computer-generated face. Overall, the mean estimated age was 26.09 (*SD*=4.50) with a mode of 25 years. The mean estimated age in the younger group was 25.10 years old (*SD* = 3.65), while the older group’s mean estimated age of the computer-generated face was 27.10 years (*SD* = 5.04). This difference was significant according to a one-tailed independent samples t-test; *t*(220.39) = 3.56, *p* < 0.001, Cohen’s *d* = .46. A one-tailed Pearson correlation was computed between participants’ own age and their age estimates for the computer-generated face. The relationship was not found to be significant: *r*(244) = 0.10, *p =* 0.05. This suggests that the own-age anchor effect does not readily generalize to all faces.


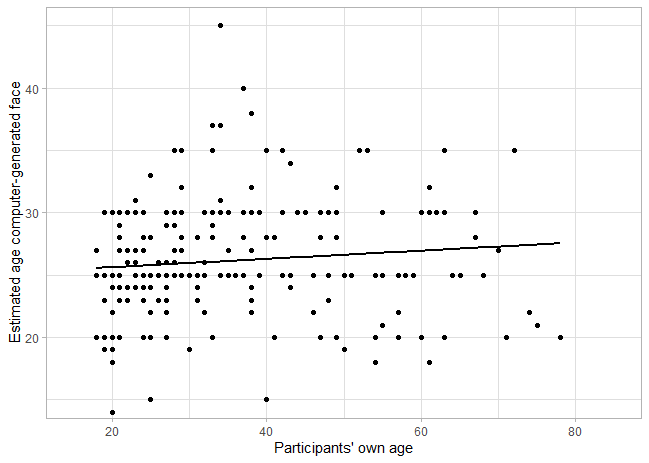


**Figure S3.** Relationship between participant’s own age and the age they estimated the computer-generated face to be.

In comparison with the age estimates for the ‘my wife/mother-in-law’ figure, the spread of the age estimates for the computer-generated face is notably smaller. Therefore, the correlation between own age and estimated age is likely to have been reduced by a restriction of the range of the age estimates. Estimating the age of a face that was generated to be representative of the average 30-year-old Caucasian female might have been fairly easy for participants. The own-age anchor effect might not present for this particular stimulus because the age information contained in the face might be sufficiently clear for participants to make an accurate age estimate, even when it is only briefly presented. The observation that the age estimates are indeed close to the intended age of the face supports this interpretation. Future research in to the own-age anchor effect might want to use more ambiguous or more naturalistic stimuli, as the effect is most likely to present under uncertain conditions (Clifford et al., 2018).

1. References

Clifford, C. W. G., Watson, T. L., & White, D. (2018). Two sources of bias explain errors in facial age estimation. *Royal Society Open Science, 5*(10), 180841. http://dx.doi.org/10.1098/rsos.180841

Nicholls, M. E. R., Churches, O., & Loetscher, T. (2018). Perception of an ambiguous figure is affected by own-age social biases. *Scientific Reports*, *8*(1). https://doi.org/10.1038/s41598-018-31129-7

R Core Team R. (2019). *A language and environment for statistical computing.* https://www.R-project.org/.
